# National Confederation of State Medical Examining and Licensing Boards

**Published:** 1897-07

**Authors:** 


### NATIONAL CONFEDERATION OF STATE MEDICAL EXAMINING AND LICENSING BOARDS.

THIS organisation held its seventh annual meeting at the Hotel Walton, Philadelphia, May 31, 1897. The meeting was called to order at 10 o'clock A. M., and remained in session until 6.40 P. M., excepting a short noon recess. The roll call showed twenty-five states and territories represented by official delegates, besides a large number of individual members. The University of the State of New York was represented officially in the person of one of its regents, the Hon. T. Guilford Smith, of Buffalo.

An address of welcome was made by Dr. A. H. Hulshizer, member of the Pennsylvania state board of medical examiners. He referred to the status of Pennsylvania concerning medical education, and especially dwelt upon the fact that Philadelphia is a medical center and a city full of professional and historic interest. His well-rounded periods were received with applause. The response was made by one of the vice-presidents, Dr. Charles A. L. Reed, of the Ohio state board of registration and examination. Dr. Reed's wonted eloquence and rhetoric were displayed to good advantage on this occasion, and the comprehensiveness with which he surveyed the field of medical educational reform, as well as his polite reference to Dr. Hulshizer's address, received the plaudits of the assemblage. Regent T. Guilford Smith, of the university of the state of New York, followed with a brief address, in which he manifested an interest and knowledge of questions relating to medical education that even surprised many of the doctors. He showed that the university which he represented was in touch with the sentiment of the age on these questions, and stood ready to meet any reasonable proposition having for its object the general welfare. Regent Smith's remarks were well received and generously applauded.

The secretary and treasurer, Dr. A. Walter Suiter, of Herkimer, then read his report, which was referred to the executive council to be audited and to receive such other action as might be deemed essential. The president, Dr. William Warren Potter, of Buffalo, next delivered his address, which will be found published in full in another column, page 883. A recess was then taken until 2.30 o'clock.

When the confederation met in the afternoon, Dr. J. W. Holland, Dean of Jefferson Medical College, addressed the assemblage in a felicitous manner. He presented thoughts, from the standpoint of the teacher relating to state examinations, that evinced a growing interest among the colleges on the subject, and that put the speaker at once in touch and sympathy with the examiners. Dr. Holland's scholarly effort was accorded hearty cheers at its conclusion.

The acting chairman of the committee on minimum standards, Dr. N. R. Coleman, of Ohio, then presented the preliminary report of his committee. The underlying feature of this report was the recommendation that a high school course should be adopted as the minimum of the equipment of a student for entrance upon the study of medicine; but inasmuch as there is great variation in rules among the states as to what constitutes a high school course, Dr. Coleman submitted a curriculum which approaches the New York and Michigan high school standards. The consideration of this report developed a spirited debate, that was participated in by many prominent speakers from many different sections of the Union. Regent T. Guilford Smith, in the course of his remarks on the report, said that New York would undoubtedly be willing to meet other sections of the country on equitable grounds, and would make any reasonable concession that might lead to the universal establishment of a minimum standard of requirements suited to the present conditions existing in the several states. This sentiment met with hearty approval, and the confederation greeted it with applause. Dr. Coleman closed the debate in an able and spirited address, that demonstrated his familiarity with the subject. The report was then referred back to the committee, which was continued and directed to report next year.

Dr. William S. Foster, of Pittsburg, secretary of the Pennsylvania state medical examining board, presented a paper entitled,

Practical experience with, and results of, the medical law of Pennsylvania; and Dr. W. H. Sanders, of Montgomery, Ala., followed with a paper entitled, The Alabama system. These interesting papers were then discussed by several members who accentuated the salient features of the essays. Dr. Charles K. Cole, of Helena, Montana, sent a paper entitled, Remarks on medical practice laws in the new Northwest, that was read by title and ordered printed in the proceedings. Dr. Cole's absence was very much regretted, as his experience has been large and his counsel is valuable.

The election of officers then took place, with the following result: president, William Warren Potter, Buffalo; vice-presidents, E. L. B. Godfrey, Camden, N. J., and William Bailey, Louisville, Ky.; secretary and treasurer, A. Walter Suiter, Herkimer, N. Y. The executive council and the committee on minimum standard of requirements were continued and the president directed to fill vacancies.

The meeting room was a most excellent one and was supplied through the courtesy of Mr. I. D. Crawford, manager of the Hotel Walton. A resolution thanking Mr. Crawford for this and many other pleasant attentions to the Confederation was passed, some other miscellaneous business transacted, and at 6.40 P. M. the Confederation adjourned, after having spent a busy and profitable day, in which much progress was made in the direction of unifying, methodising and standardising the work of the state boards of medical examiners throughout the United States.

---

Dr. George Ben Johnston, of Richmond, president of the Medical Society of Virginia, was chosen by the committee on jubilee exercises to speak for the state medical societies. He delivered a brief and telling address in the following words:

### WHAT THE STATE MEDICAL SOCIETIES HAVE DONE.

The enactment of such laws as now exist was, it is fair to claim, procured through the efforts of the state societies acting independently of each other. There was never any well-directed attempt at coöperation, hence the dissimilar character of the various statutes and the varying nature of the prescribed machinery for executing them. Yet in every case the object was the same—to furnish the people with only properly equipped practitioners. This called into existence many state examining boards, whose function was to select qualified men. It was soon made apparent that even many of those holding diplomas were not qualified. Careless and inferior schools saw their graduates excluded from the privileges of practice and their classes diminished, because young men contemplating the study of medicine began to choose their schools with discrimination, having in view the purpose of properly fitting themselves to meet the requirements of the examining boards. The effect of all this was to elevate the standard of medical education. New subjects were added to the college curriculums, practical work was required, the time of study lengthened, and more rigid examinations exacted.

It became obvious that these legislative measures to secure higher medical education usually styled Laws regulating the practice of medicine and surgery—they would be more accurately described as Laws for the protection of the people—had taken root and had come to stay. National bodies have taken them up and are rapidly centralising the work accomplished in a desultory but partially effective way by the state societies. On Monday of this week we witnessed in this city the seventh annual meeting of the National Confederation of State Medical Examining and Licensing Boards. This association is the legitimate and natural outgrowth of the efforts of the individual societies which have for so many years patiently but unconsciously worked for its formation and development. Although as yet it is scarcely more than embryonic, its function is well defined, and it must become the leader in further efforts to secure appropriate and uniform laws regulating the practice of medicine, and state societies ought to earnestly coöperate with it in order that it may be strengthened and its usefulness increased. But the results obtained in another department of legislation are not less striking.

In most of the states in which state boards of health have been established, these bodies have been brought into existence through the suggestion and instrumentality of the state medical societies. These boards have been of inestimable value to the public in the way of promulgating the laws of hygiene, in the prevention of epidemics, the suppression of those already existing, and the conduct of commerce in times of public fear.

If then, as I believe, the state societies were the main instruments in calling into existence the State boards of medical examiners and licensers, and the state and other boards of health, may we not justly claim for them the title of public benefactors? They fallowed the field and sowed the seed; the community is reaping the harvest. And how are their labors made manifest?

By the improvement in the material from which medical men are formed. By the better preliminary education of medical students.

By the higher technical education of doctors.

By the binding together of the best medical colleges in a league, the sole object of which is to elevate the standard of medical education.

By the enactment of laws which admit only properly equipped men to enter the practice of medicine.

By the rapid disappearance of the pretender and the ignorant quack.

By the presence in every village or hamlet of a medical man whose character and attainments entitle him to rank with the best of his community; whose professional acumen and honesty fit him to discharge the duties of his high calling with confidence, skill and success, and whose trained faculties constitute him the guardian of the public health.

All these beneficent results may be traced to the working of your state societies.

